# Effect of storage temperature and produce type on the survival or growth of *Listeria monocytogenes* on peeled rinds and fresh-cut produce

**DOI:** 10.3389/fmicb.2023.1151819

**Published:** 2023-06-15

**Authors:** Juan Moreira, Erika Mera, Vijay Singh Chhetri, Joan M. King, Thanos Gentimis, Achyut Adhikari

**Affiliations:** ^1^School of Nutrition and Food Sciences, Louisiana State University AgCenter, Baton Rouge, LA, United States; ^2^College of Agriculture and Food Sciences, Florida A&M University, Tallahassee, FL, United States; ^3^Department of Experimental Statistics, Louisiana State University, Baton Rouge, LA, United States

**Keywords:** *Listeria monocytogenes*, die-off, fresh cut produce, contact angle, contamination

## Abstract

Whole and fresh-cut produce are minimally processed and susceptible to microbial contamination. This study evaluated the survival or growth of *L. monocytogenes* on peeled rinds, and fresh-cut produce at different storage temperatures. Fresh-cut fruits and vegetables, including cantaloupe, watermelon, pear, papaya, pineapple, broccoli, cauliflower, lettuce, bell pepper, and kale (25 g pieces) were spot inoculated with 4 log CFU/g of *L. monocytogenes* and stored at 4 or 13°C for 6  days. Cantaloupe and bell pepper rind disks (20 cm^2^), mimicking whole produce were inoculated with low inoculum level (4 log CFU/mL) and high inoculum level (6 log CFU/mL) and stored at 24°C up to 8  days and 4°C up to 14  days, respectively. *L. monocytogenes* counts on fresh-cut pear samples stored at 4°C increased significantly by 0.27 log CFU/g. However, Listeria levels on kale (day 4), cauliflower (day 6), and broccoli (day 2) were significantly reduced by 0.73, 1.18, and 0.80 log CFU/g, respectively, at 4°C. At 13°C, the bacterial counts increased significantly after a day of storage on fresh-cut watermelons (increasing by 1.10 log CFU/g) and cantaloupes (increasing by 1.52 log CFU/g). Similar increases were observed on pears (1.00 log CFU/g), papayas (1.65 log CFU/g), and green bell peppers (1.72 log CFU/g). Pineapple samples did not support the growth of *L. monocytogenes* at 13°C with a significant reduction of 1.80 log CFU/g by day 6. *L. monocytogenes* levels significantly increased in fresh-cut lettuce at 13°C but remained stable on kale, cauliflower, and broccoli after 6  days of storage. Stable population was observed also on cantaloupe rinds up to 8  days at 24°C. While on the outer surface of bell peppers, the population level decreased below the detectable limit of the test (10 CFU/20 cm^2^) after 14  days of storage at 4°C. The results demonstrated variable survival behavior of *L. monocytogenes* on fresh-cut produce with produce type and storage temperature.

## 1. Introduction

Microbial risk in fresh produce begins during production and harvesting at the farm level (Barton et al., 2015). At the farm, there is a risk of exposure to sources of contamination, such as irrigation water, animals, and soil (Allende et al., 2015; Amoah et al., 2015). After contamination events, microbial pathogens initially attach to the surface of produce. At this initial stage microorganisms are loosely attached and a significant population could be removed by cleaning and sanitation procedures (Fletcher et al., 2018). However, with a lack of proper sanitation and unfavorable growth environments these microorganisms can produce protein-based protective covers known as biofilms. Several intrinsic/extrinsic factors such as produce surfaces, changes in pH, water activity, and temperature can affect the survival and attachment of bacteria. Once biofilm formation occurs bacteria are considered firmly attached to a surface, making it difficult to remove contamination (Barbuddhe et al., 2015; Briandet et al., 2015; Fletcher et al., 2018).

Minimally processed produce depends mostly on natural barriers such as peels and rinds to protect from bacteria infecting the inner mesocarp. However, during slicing or cutting pathogens may transfer into the flesh. The nutrients present in the produce may support the survival and growth of pathogens increasing produce safety risks (Kumar et al., 2015; Bortolossi et al., 2016). Contact angle and surface free energy are important parameters used to evaluate the interactions between liquid and solid surfaces and their potential for microbial attachment. These two components together can determine the hydrophobic or hydrophilic properties, by measuring the wettability and adhesion between a solid and a liquid (Adhikari et al., 2015; Aryal et al., 2023). These conditions, along with its ability to grow at refrigeration temperatures, aid *L. monocytogenes* in attaching to fresh-cut produce as well as on other environments, such as surfaces in food processing plants (East et al., 2016). Pathogens are present in low numbers in an agricultural production environment which is why the sensitivity and specificity of the test is crucial to accurately evaluate the microbial risk associated with fresh produce. Guidance document such as EURL Lm Guidance Document provide scientific methods for shelf life studies and challenge test specific to *L. monocytogenes* on ready to eat foods (Bergis et al., 2021). Our previous study indicated that bacterial pathogens exposed to environmental conditions may develop potential to overcome post-harvest treatment conditions. Studies considering environmental factors during production, processing and at the point of display would provide scientific data to precisely evaluate the risk and revise the validation and verification procedure.

*Listeria monocytogenes* is of importance to public health because of its high mortality rate (20%) and potential to cause listeriosis in immunocompromised individuals. Listeriosis in its gastroenteric and non-maternal-neonatal form can lead to mild symptoms such as fever, nausea and diarrhea or severe symptoms which include septicemia and meningitis (Bergis et al., 2021). Maternal-neonatal listeriosis is of higher concern for pregnant women due to its ability to lead to miscarriages (Kljujev et al., 2018). Cross-contamination may occur at several points during post-harvest conditioning. As stated by Dalmasso et al. (2015), food-processing environments are affected by three types of possible contamination. The first being sporadic contamination, which involves contact between unclean and clean surfaces. This contamination frequently occurs during the receiving of raw materials from the field, which has a greater potential of carrying microbial pathogens. Another possible contamination scenario is hotspots of contamination; this refers to areas in which improper cleaning has led to the build-up of waste and in many cases, biofilm formation of *L. monocytogenes*. Moreover, *L. monocytogenes* and its ability to attach to different produce and food-contact surfaces makes it one of the main concerns in these situations. These hotspots are difficult to identify. Due to high microbial populations, produce is at a higher risk of contamination. The final scenario is widespread contamination, caused mainly by improper cleaning protocols, which allow isolated contaminations to spread throughout most of the food-processing environment (Dalmasso et al., 2015). In order to reduce risk of contamination during post-harvest processing it is necessary to apply proper sanitizing procedures for the produce, as well as proper and constant sanitizing protocols for the entire food-processing facility, as to minimize the possibility that the environment becomes a vector of contamination (Allende et al., 2015).

The contamination of fresh produce with *L. monocytogenes* has frequently been reported through pre-and post-harvest activities with processing plants/facilities as one of the major routes of contamination (Caleb et al., 2014; Gerba et al., 2014; Holvoet et al., 2014; Barbuddhe et al., 2015; Dalmasso et al., 2015). During an outbreak involving whole cantaloupes from Colorado contaminated with *L. monocytogenes* during 2012, a total of 143 people were hospitalized, 33 deaths were reported and the outbreak spanned throughout 28 different states (CDC, 2012). Two different outbreaks related to packaged salads in the United States in 2016 and 2022, accounted for 19 and 10 hospitalizations, respectively (CDC, 2016; CDC, 2022). Once *L. monocytogenes* enters into the processing facilities, it can survive for long periods due to favorable humidity and temperature (Dalmasso et al., 2015). Studies have reported mixed results on the survival of *L. monocytogenes* on fresh and cut produce, with the pathogen having successfully internalized in some produce and in other cases having been unable to survive throughout its shelf-life. The disparities between the findings may be attributed to produce surface characteristics and available nutrients (Gerba et al., 2014), and results based on a single variety, may not represent all produce. Furthermore, a majority of studies used the inoculum levels of 8 or 9 log CFU/g in their studies, which may be higher than real world situations. These discrepancies make it difficult to relate behaviors of pathogens such as *L. monocytogenes*, to actual outbreak scenarios. Most of the outbreaks usually arise from a much lower level of contamination. Moreover, there are knowledge gaps on the influence of contamination level and the storage temperature on the survival behavior of *L. monocytogenes* on produce surfaces. Therefore, the objective of this study was to examine the growth or survival of *L. monocytogenes* on produce under different storage temperatures.

## 2. Materials and methods

### 2.1. Inoculum preparation

A cocktail of four strains of Nalidixic acid resistant *L. monocytogenes* [LCDC 81–861 (4b), V7 (1/2a), 101 M (4b), and Scott A (4b)] obtained from Dr. Michelle Danyluk, University of Florida were used in this study in order to have a representative sample of *L. monocytogenes* and its most virulent serotypes (4b and 1/2a). Nalidixic acid resistant mutants of all four strains were developed by culturing in media supplemented with Nalidixic Acid until each strain was resistant to a concentration of 0.05 mg/ml of this antibiotic, Frozen cultures were activated in three successive passes and concentrated to an inoculum of 8 log CFU/ml. Briefly, the frozen cultures were thawed, vortexed (Fischer Scientific vortex), and 0.1 ml was transferred into 10 ml test tubes of Tryptic Soy Broth (Criterion) containing 0.6% yeast extract (TSBYE) (VWR Inc. USA). The TSBYE test tubes were supplemented with Nalidixic Acid at a concentration of 0.05 mg/ml and were later incubated at 37°C for 24 h. After incubation, 1 ml was transferred into a fresh 10 ml test tube of TSBYE and incubated at 37°C for 24 h. The process was repeated with 0.1 ml inoculum to another fresh 10 ml test tube of TSBYE and incubated at 37°C for 24 h. Cells were then harvested after 24 h by centrifugation (Labnet Spectrafuge 6C Centrifuge) at 6500 rpm for 5 min. The supernatant was discarded, and 10 ml of 1x Phosphate Buffer Saline (PBS) was added to the pellet. The cocktail of four *L. monocytogenes* strains was prepared by mixing each in equal volume and adjusting a cell density of 8 log CFU/ml. The cocktail was then serially diluted to 4 log CFU/ml for the low inoculum level study and 6 log CFU/ml for the high inoculum level study as described in previous studies, the inoculum was then kept inside a laminar flow biosafety hood until used (Kharel et al., 2018; Chhetri et al., 2019).

### 2.2. Sample preparation

Mature fruits (Sol Group cantaloupes, pears, honey seeded variety watermelons, papayas var. Maradol and pineapples var. Piña Miel) and vegetables (lettuce, kale, cauliflower, broccoli, and green bell peppers) were purchased from local wholesale markets (Southside Produce Co.) and stored in the refrigerator at 4°C overnight.

In order to mimic whole cantaloupes and bell peppers, circular rinds (20 cm^2^) were cut out of these samples using a sterilized knife and the internal mesocarp was removed. Fresh-cut samples were prepared by cutting fruits or vegetables into cubes of 4 × 4 cm^2^ (weight 25 g) with a sterile knife. Each replicate was prepared from two cantaloupes, three papayas, two pineapples, six pears, and one watermelon for fruit samples. For vegetable samples each replicate was prepared from approximately, six green bell peppers, two cauliflower heads, two broccoli heads, one lettuce head and a 500 g bag of kale. Additional samples of fresh-cut fruits and vegetables were prepared in order to determine the pH of the samples, which was measured during the first and last day of the analysis using a H30PCO Multi-Parameter Handheld Meter (VWR Inc. USA) after homogenizing samples in a Bagmixer 400 blender (Interscience Laboratories Inc., USA).

### 2.3. Inoculation and storage conditions of samples

Rind samples of cantaloupes and green bell peppers were spot inoculated with 0.5 ml of either low (4 log CFU/ml) or high (6 log CFU/ml) inoculum. Fresh-cut fruit and vegetables under different storage conditions were spot inoculated with 0.5 ml of low (4 log CFU/ml) inoculum. Samples were left to dry inside the biosafety laminar flow hood for 1 h. Afterward, samples were transferred to either 4, 13, or 24°C storage conditions. Storage temperature of 4°C was used to represent refrigerated storage conditions, and 13°C was used to represent temperature abuse conditions, meanwhile 24°C was used to represent average room temperature. Fresh-cut samples were stored at 4 or 13°C for 6 days, green bell pepper rind samples were stored at 4°C for 14 days, and cantaloupe rinds were stored at 24°C for 8 days. Storage temperatures were established by considering the regular temperatures at which these products would be stored by consumers. For example, a whole cantaloupe is commonly stored outside of refrigeration, meanwhile fresh-cut cantaloupe is stored at refrigeration temperatures (4 or 13°C). Likewise, the duration of the storage of each sample was established by considering the common shelf-life of this product, while it is stored in the commonly used temperature, and then doubling that time. For example, cantaloupe rinds were stored at 24°C for 8 days, considering a regular shelf-life of 4 days.

### 2.4. Sample analysis

Rind samples were removed from incubation, and the uppermost layer was peeled using sterilized peelers. Afterwards the peeled layer placed in a Fisher brand centrifuge tube using sterile tweezers. The sample was then weighed and the weight of each was recorded. Afterward 20 mL of DE Neutralizing Broth (Hardy Diagnostics, OH, USA) was added to the centrifuge tube and with a sterile scissor, the sample was cut into smaller pieces. A vertical Fisher Scientific 150 Handheld Homogenizer was used to homogenize the sample. Later 1 ml of the sample was extracted, and it was diluted accordingly in 9 ml test tubes of 0.1% peptone water. Samples were then plated in duplicate on Oxford *Listeria* Agar (Hardy Diagnostics, OH, USA) supplemented with Nalidixic Acid at a concentration of 0.05 mg/ml. These plates were then incubated at 37°C for 24 h with round colonies with a black halo around them being counted as *L. monocytogenes*.

Fresh-cut samples were removed from incubation and placed inside a biosafety hood in which the pH was determined for samples corresponding to the first and last day of analysis, by homogenizing the additional samples and then using a VWR H30PCO Multi-Parameter Handheld Meter. Each sample was then transferred to Nasco Whirl-Pack Filter Bags, and the weight was recorded. Then 100 ml of DE Neutralizing Broth was added to the whirl-pack filter bag, and the bag was stomached for 5 min using Interscience Bag Mixer SW. The sample was then diluted accordingly in 9 ml test tubes of 0.1% Peptone Water, and 0.1 mL was plated in duplicate on Oxford *Listeria* Agar Plates supplemented with Nalidixic Acid at a concentration of 0.05 mg/ml. The plates were then incubated at 37°C for 24 h in a Sanyo MCO-20AIC CO_2_ Incubator. Yeast and Mold, and Aerobic Plate Count 3 M Petri films were plated with the diluted samples and incubated on the first and last day of analysis for fresh-cut samples, according to the manufacturer’s specifications. Yeast and Mold counts consisted of the total of yeast and mold colonies found present on the fruit or vegetable sample analyzed. All the analysis carried out in this study were replicated in three different periods of time, in order to have appropriate replication for each fruit or vegetable fresh-cut, and rind samples.

After 24 h of incubation at 37°C, the *L. monocytogenes* population on rind samples was counted and the number was converted to log CFU/cm^2^, by determining the population in CFU/20 cm^2^ disk and dividing by 20. Rind samples with the count below the detectable limit (<10 CFU/g) were enriched in Tryptic Soy Broth with Yeast Extract and incubated at 37°C for 24 h and then plated on Oxford *Listeria* Agar plates supplemented with Nalidixic Acid at 37°C for 24 h, as a qualitative test in order to determine presence or absence of *L. monocytogenes*. For fresh-cut samples, the count was converted to log CFU/g. Yeast and Mold Petri films were counted every 24 h up to three-day while Aerobic Plate Count Petri films were counted after 24 and 48 h.

### 2.5. Contact angle and surface free energy determination

Contact angle measurements were performed with a sessile drop method using a FTA1000 Analyzer System with an Edmund Optics 55,382 platform and a Firefly MV-03TM camera (First Ten Angstroms, USA) at 25°C. A microliter syringe equipped with a 0.5 mm diameter needle was used to dispense approximately 0.5–1.0 μl of either a polar liquid (double-distilled water) or a nonpolar liquid (diiodomethane of 99% purity, Sigma Aldrich) onto fruit or vegetable samples of approximately 1 mm of thickness. The contact angle was measured immediately upon coming in contact with the surface, and this was repeated in triplicate using new samples each time. Surface free energy of the fruits and vegetables analyzed was determined with Fowkes equation (Adhikari et al., 2015):


(1) $$\sqrt{\sigma_{l}^{D}*\sigma_{S}^{D}}+\sqrt{\sigma_{l}^{P}*\sigma_{S}^{P}}=\frac{\sigma_{l}*\left(1+\cos\theta \right)}{2}$$

Where ϴ is the contact angle measurement in degrees, σ_l_ is the surface tension in mN/m, σ^D^ is the dispersion component (mN/m) and σ^P^ is the polar component (mN/m) of either liquid (l) or solid (s).

### 2.6. Statistical analysis

The experimental design for this study consisted of a Completely Randomized Design, comparing microbial counts of the same bacteria between timepoints for the same matrix. The data was analyzed using Statistical Analysis Software 9.4, with a post ANOVA, Tukey mean separation and a LS Means in order to calculate the statistical significance of differences in between observations. A *p*-value <0.05 was considered statistically significant.

## 3. Results

### 3.1. Survival or growth of *Listeria monocytogenes* in fresh-cut fruits

**Figure 1:**
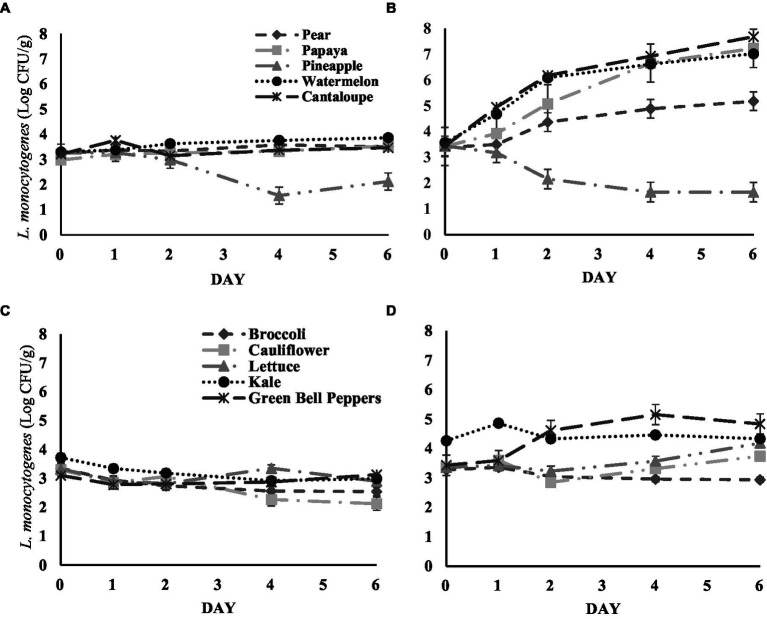
**(A)** Survival of *L. monocytogenes* inoculated on fresh-cut fruit samples stored at 4°C. **(B)** Fresh-cut fruit samples stored at 13°C. **(C)** Survival of *L. monocytogenes* inoculated on fresh-cut vegetable samples stored at 4°C. **(D)** Fresh-cut vegetable samples stored at 13°C. Analysis for each fruit or vegetable sample was carried out in triplicate.

**Figure 2:**
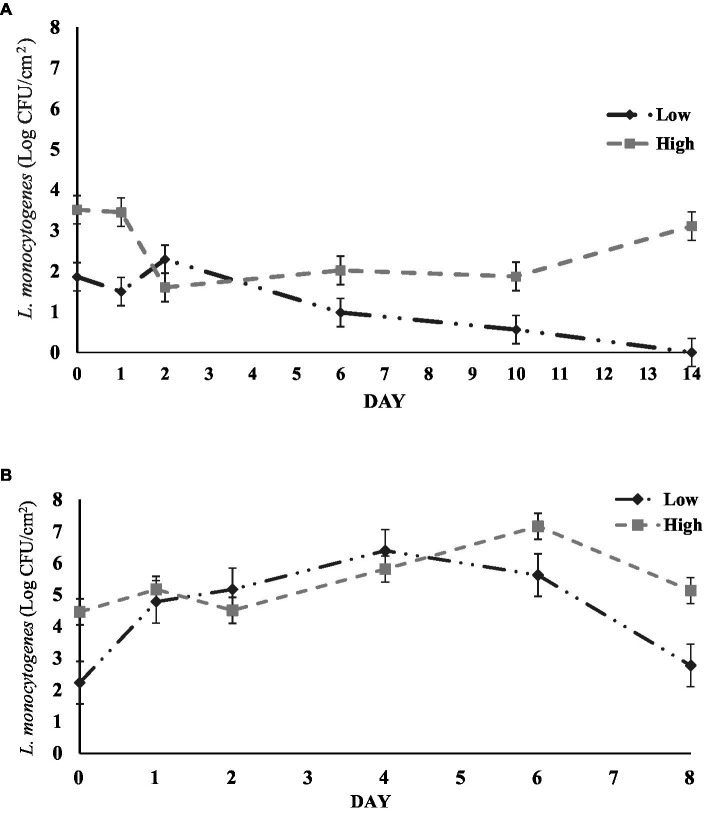
**(A)** Survival of *L. monocytogenes* inoculated on cantaloupe rind samples at 24°C. Low: low inoculum level, High: high inoculum level. **(B)** Survival of *L. monocytogenes* inoculated on bell pepper rinds at 4°C. Low: low inoculum level, High: high inoculum level. Analysis for each either cantaloupe or bell pepper rind samples were carried out in triplicate.

The growth or survival of *L. monocytogenes* on five different fresh-cut fruit samples during storage is shown in Figures 1A,B. At 4°C storage fresh-cut fruits (Figure 1); cantaloupe, papaya, watermelon, and pineapple, did not present significant changes in *L. monocytogenes* populations. Pear samples stored at the same storage temperature presented significant growth of *L. monocytogenes*, with a 0.34 log CFU/g increase by day 4 (3.58 ± 0.15 log CFU/g) in comparison from the initial population (3.24 ± 0.06 log CFU/g) (*p* < 0.05). At 13°C, significant growth of *L. monocytogenes* was observed on all fresh-cut fruits after 2 days of storage, except on pineapple samples (Figure 2). *L. monocytogenes* was significantly reduced by 1.80 log CFU/g in pineapple samples by the last day at 13°C storage (1.64 ± 0.10 log CFU/g) (*p* < 0.05). The *L. monocytogenes* showed a significant growth on watermelons and cantaloupes on day 2, by 3.45 log and 4.24 log CFU/g, respectively (*p* < 0.05). On pears and papaya, a significant increase in the populations were observed on day 2, by 1.79 log CFU/g and 3.80 log CFU/g, respectively (*p* < 0.05). Among the tested fresh-cut produce, the highest growth was observed on cantaloupe followed by papaya, watermelon, and pears. The pH of all fresh-cut fruit or vegetable samples was not significantly different from the first and last day of analysis, therefore it was not considered a significant factor in the growth of *L. monocytogenes* (Supplementary Tables S1, S2).

### 3.2. Survival or growth of *Listeria monocytogenes* in fresh-cut vegetable samples

The growth or survival of *L. monocytogenes* on five different fresh-cut vegetable samples during storage is shown in Figures 1C,D. *L. monocytogenes* populations decreased with time on broccoli, and cauliflower at storage temperature of 4°C up to day 2 of analysis. At 6 and 2 days of storage, respectively, *L. monocytogenes* was significantly reduced on cauliflower (by 1.03 log CFU/g) and broccoli samples (by 0.78 log CFU/g) (*p* < 0.05). The bacterial population of *L. monocytogenes* on kale was significantly reduced by 0.73 log CFU/g on day 6 of storage when compared to the initial population (*p* < 0.05).

Fresh cut vegetables stored at 13°C showed mixed results for *L. monocytogenes* population (Figure 1D). *L. monocytogenes* levels remained similar on broccoli, cauliflower, and kale samples during storage at 13°C with no significant changes detected between days of analysis. While on green bell peppers the *L. monocytogenes* count increased significantly, by 1.72 log CFU/g after the fourth day of storage (*p* < 0.05). Similarly, on lettuce samples, the population increased significantly by 0.82 log CFU/g after 6 days of storage (*p* < 0.05). Similarly, to fresh cut fruits, the pH of all fresh-cut vegetables was not significantly different from the first and last day of analysis.

### 3.3. Survival or growth of *Listeria monocytogenes* on cantaloupe and green bell pepper rinds

The changes in *L. monocytogenes* population on cantaloupe rinds and bell pepper rinds during storage at 24°C is shown in Figures 2A,B. We observed a similar survival pattern between low and high inoculum samples over time. For cantaloupe rinds, the initial *L. monocytogenes* count on low inoculum samples was 2.23 log CFU/cm^2^, which increased significantly by day 4–6.39 log CFU/cm^2^ (*p* < 0.05). However, the population declined in the subsequent days and decreased significantly to 2.77 log CFU/cm^2^ on day 8 (*p* < 0.05). A similar survival trend was observed on high inoculum samples from initial levels 4.46 log CFU/cm^2^, to 7.16 log CFU/cm^2^ on day 6. However, the bacterial level decreased significantly after day 6 to 5.13 log CFU/cm^2^ (*p* < 0.05).

For bell pepper, the *L. monocytogenes* population on both high inoculum and low inoculum samples remained the same overtime during storage at 4°C. The initial count on high inoculum samples was 3.51 log CFU/cm^2^ and did not significantly change throughout the duration of the study (Figure 2B). On low inoculum samples, the population did not present any significant changes during the study. However, the population decreased below the detectable limit after 14 days. After carrying out the qualitative enrichment tests on these samples, the tests showed no growth of the pathogen indicating that *L. monocytogenes* was either not present or none culturable on the surface of green bell peppers.

### 3.4. Mesophilic aerobic bacteria and yeast and mold present on fresh-cut fruits and vegetables

The mesophilic aerobic bacterial populations on fruit samples during storage at 4 and 13°C are shown in Tables 1A,B, respectively. Bacterial populations at the 4°C storage temperature was significantly higher on the final day of storage, compared to the first day (*p* < 0.05). On average MAB populations increased a total of 2.24 log CFU/g from the first day to the last day of analysis (*p* < 0.05). MAB populations of fresh-cut fruit samples stored at 13°C did not change significantly from the first day up to the last day of analysis, with the exception of MAB on papaya samples. MAB increased in papaya samples from 4.00 log CFU/g on the first day to 4.66 log CFU/g (*p* < 0.05). The yeast and mold population present on fresh-cut fruits stored at 4 or 13°C are shown in Tables 2A,B, respectively. Yeast and mold levels on pear and cantaloupe samples remained similar but increased significantly on papaya, pineapple and watermelon samples stored at 4°C (*p* < 0.05). Fresh-cut fruit samples stored at 13°C did not see any significant changes in yeast and mold populations during the study.

**Table 1:** Aerobic bacteria population (Log CFU/g) present on surface of fresh-cut fruit samples on the first and last day of storage at **(A)** 4°C or **(B)** 13°C storage temperature.

A						
	Fruits					
Storage temperature	Day	Pear	Papaya	Pineapple	Watermelon	Cantaloupe
4°C	0	3.43 ± 0.99	3.22 ± 0.54	2.60 ± 0.59	3.06 ± 0.59	3.38 ± 0.42
4°C	6	6.00 ± 1.02^*^	6.02 ± 0.45^*^	4.36 ± 1.34^*^	5.62 ± 0.62^*^	5.28 ± 0.68^*^
B						
		Fruits				
Storage temperature	Day	Pear	Papaya	Pineapple	Watermelon	Cantaloupe
13°C	0	3.78 ± 0.24	4.00 ± 0.24	3.24 ± 0.18	4.62 ± 0.24	4.26 ± 0.11
13°C	6	4.30 ± 0.20	4.66 ± 0.54^*^	3.84 ± 0.04	5.73 ± 0.10	4.93 ± 0.05

Means in the same column for each temperature with * are significantly different (*P* < 0.05). Analysis of each fruit at either 4 or 13°C storage temperature was carried out in triplicate.

**Table 2:** Yeast and Mold population (Log CFU/g) present on surface of fresh-cut fruit samples on the first and last day of storage at **(A)** 4°C or **(B)** 13°C storage temperature.

A						
	Fruits					
Storage temperature	Day	Pear	Papaya	Pineapple	Watermelon	Cantaloupe
4°C	0	3.10 ± 1.92	1.76 ± 1.09	3.87 ± 2.39	2.07 ± 1.45	3.18 ± 0.55
4°C	6	4.22 ± 1.98	2.92 ± 1.71^*^	5.10 ± 1.16^*^	3.75 ± 0.26^*^	3.95 ± 0.59
B						
	Fruits					
Storage temperature	Day	Pear	Papaya	Pineapple	Watermelon	Cantaloupe
13°C	0	3.36 ± 0.06	2.93 ± 0.13	3.37 ± 0.07	3.59 ± 0.05	3.31 ± 0.22
13°C	6	4.07 ± 0.08	3.48 ± 0.09	3.97 ± 0.14	4.39 ± 0.05^*^	4.01 ± 0.28

Means in the same column for each temperature with * are significantly different (*P* < 0.05). Analysis of each fruit at either 4 or 13°C storage temperature was carried out in triplicate.

The mesophilic aerobic bacterial population on fresh cut vegetables during storage at 4 and 13°C are shown in Tables 3A,B, respectively. At 4°C storage temperature, the MAB count remained similar, with the exception of bell peppers. MAB populations on bell peppers increased significantly from 3.38 log CFU/g on the first day to 5.28 log CFU/g on the last day of analysis (*p* < 0.05). Fresh-cut vegetable samples stored at 13°C did not present significant changes in MAB populations from the beginning of the study to its end, with the exception of kale samples. The MAB population on kale samples stored at 13°C increased significantly from 6.39 log CFU/g to 6.77 log CFU/g by the end of the study (*p* < 0.05). The yeast and mold population present on fresh-cut vegetables stored at 4 or 13°C is shown in Tables 4A,B, respectively. Yeast and mold populations in fresh-cut kale and lettuce samples stored at 4°C remained similar, however, on broccoli the level increased significantly by 0.71 log CFU/g and on bell peppers by 0.82 log CFU/g (*p* < 0.05).

**Table 3:** Aerobic bacteria population (Log CFU/g) present on surface of fresh-cut vegetable samples on the first and last day of storage at **(A)** 4°C or **(B)** 13°C storage temperature.

A						
		Vegetables				
Storage temperature	Day	Broccoli	Cauliflower	Lettuce	Kale	Green bell pepper
4°C	0	4.99 ± 1.18	4.66 ± 1.62	5.46 ± 0.91	6.61 ± 0.33	3.38 ± 0.62
4°C	6	5.22 ± 0.15	4.81 ± 0.10	5.21 ± 0.08	6.33 ± 0.34	5.28 ± 0.51^*^
B						
	Vegetables					
Storage temperature	Day	Broccoli	Cauliflower	Lettuce	Kale	Green Bell Pepper
13°C	0	5.24 ± 0.89	5.23 ± 0.55	5.42 ± 0.52	6.39 ± 0.20	4.26 ± 0.40
13°C	6	5.67 ± 0.33	5.55 ± 0.12	5.61 ± 0.23	6.77 ± 0.28^*^	4.93 ± 0.23

Means in the same column for each temperature with * are significantly different (*P* < 0.05). Analysis of each vegetable at either 4 or 13°C storage temperature was carried out in triplicate.

**Table 4:** Yeast and mold population (Log CFU/g) present on surface of fresh-cut vegetable samples on the first and last day of storage at **(A)** 4°C or **(B)** 13°C storage temperature.

A						
	Vegetables					
Storage temperature	Day	Broccoli	Cauliflower	Lettuce	Kale	Green bell pepper
4°C	0	4.64 ± 0.44	4.38 ± 1.18	4.38 ± 0.52	5.29 ± 1.00	4.58 ± 0.22
4°C	6	5.35 ± 0.22^*^	3.26 ± 0.20^*^	4.81 ± 0.43	4.82 ± 0.43	5.40 ± 0.48^*^
B						
	Vegetables					
Storage temperature	Day	Broccoli	Cauliflower	Lettuce	Kale	Green bell pepper
13°C	0	4.84 ± 0.23	4.82 ± 0.43	4.20 ± 0.77	5.14 ± 0.42	4.10 ± 0.68
13°C	6	5.60 ± 0.20^*^	4.79 ± 1.00	5.25 ± 0.50^*^	5.66 ± 0.80	4.59 ± 0.73^*^

Means in the same column for each temperature with * are significantly different (*P* < 0.05). Analysis of each vegetable at either 4 or 13°C storage temperature was carried out in triplicate.

### 3.5. Influence of surface free energy on the growth of *Listeria monocytogenes*

The contact angle of selected fruits and vegetables when they came into contact with diiodomethane ranged from 36.97 and 61.88° and did not present any significant differences (Table 5). Contact angles using distilled water were highest in cantaloupe rind samples with an average of 85.41°, this was significantly higher than the contact angles observed with broccoli (51.06°) and cauliflower (43.33°) samples. Fresh-cut fruit samples analyzed with distilled water presented significantly lower contact angles than the rest of the samples, with pear averaging a contact angle of 4.29 °, watermelon 3.90°, and cantaloupe 3.75°. The surface free energy of fresh-cut fruit samples (pear, watermelon, and cantaloupe) were significantly higher values than the rest of samples with 71.59, 71.11 and 71.03 mN/m, respectively. Meanwhile, broccoli (46.89 mN/m), fresh-cut bell pepper (43.38 mN/m), bell pepper rind (37.48 mN/m) and cantaloupe rind (25.73 mN/m) all presented statistically similar surface free energy values.

**Table 5:** Contact angle of distilled water and diiodomethane and surface free energy of select fruits and vegetables.

Fruit surface	Contact angle (*ϴ*)		Surface free energy (mN/m)
	Distilled water	Diiodomethane	
Pear (FC)	4.29 ± 3.60^*^	61.88 ± 9.39	71.59 ± 0.68
Watermelon (FC)	3.90 ± 3.55^*^	44.96 ± 10.34	71.11 ± 0.52
Cantaloupe (FC)	3.75 ± 2.60^*^	36.97 ± 5.29	71.03 ± 0.20
Cantaloupe (Rind)	85.41 ± 5.11	47.21 ± 11.80	25.73 ± 1.87^*^
Broccoli (FC)	51.06 ± 19.50^*^	54.81 ± 12.50	46.89 ± 14.22^*^
Cauliflower (FC)	43.33 ± 21.30^*^	46.81 ± 10.00	52.55 ± 14.13
Bell pepper (FC)	56.46 ± 8.41	59.11 ± 13.73	43.38 ± 6.17^*^
Bell pepper (Rind)	67.23 ± 4.04	45.05 ± 3.08	37.48 ± 2.12^*^

Means in the same column with * are significantly different (*P* < 0.05). FC, Fresh-cut; Rind, Outer peel. Analysis for each fruit or vegetable sample was carried out in triplicate.

## 4. Discussion

Refrigerated storage conditions have been shown to be an effective way to control the growth of bacteria on produce in several studies. Bai et al. (2015) found that storing papaya at 4–5°C was sufficient to stop the growth of *L. monocytogenes*. Huang et al. (2019) similarly observed an inability of *L. monocytogenes* to grow at constant refrigeration temperatures of 4–5°C, although slow population increases on the surface of fresh-cut cantaloupe and watermelon samples was observed (Huang et al., 2019). However, the results from Danyluk et al. (2014) differed from those of our study for cantaloupe samples stored at 4°C for 6 days. Danyluk et al. (2014) predicted an approximate increase of up to 1 log CFU/g at 4°C conditions, while our study observed no significant increase in fresh-cut fruits samples stored at 4°C. These differences may be due to disinfection with 200 ppm chlorine while preparing cantaloupe samples, which may resulted in minimizing natural background microflora and allowing the pathogen to grow at higher rate (Danyluk et al., 2014). In another study, Kroft et al. (2022) found that *L. monocytogenes* decreased on fresh-cut pineapple at 4, 10, and 15°C (from 2 to 3 log CFU/g decrease), while fresh-cut cantaloupe samples presented significant increases in *L. monocytogenes* population at 10 and 15°C (2.5 log CFU/g increase).

Produce characteristics may affect the survival of microorganisms. We observed a variance in *L. monocytogenes* growth and survival based on the type of fruit. Our results for fresh-cut pear samples coincide with a study from 2017, in which inoculated pear samples stored at 10°C presented an increase of 2.05 log CFU/g after 7 days of storage (Abadias et al., 2017). Similar to our study, Abadias et al. (2017) determined that cantaloupes provided an appropriate environment for the growth of *L. monocytogenes* with an increase in the population by 3.88 log CFU/g. The inability of *L. monocytogenes* to grow in pineapples could be attributed to the antimicrobials (saponin and bromelain) that may be naturally present in this fruit. One of the most likely reasons is the acidic pH in pineapple which causes bacterial pathogens such as *L. monocytogenes* to undergo cell lysis caused by the increased amount of hydrogen atoms in the environment (Dewi et al., 2018). Recent studies indicated that the enzyme bromelain, which is naturally found in pineapples, also may have antimicrobial properties (Bai et al., 2015; Dewi et al., 2018). Erfan et al. (2018) found that this compound was able to inhibit the growth of *Enterococcus faecalis*, a gram-positive bacteria. However, further studies may be needed in order to better understand the factors that make pineapple an inadequate growth medium for *L. monocytogenes.* Changes in pH of samples during storage can lead to significant changes in the growth potential of *L. monocytogenes* as it is considered one of the most important intrinsic factors during shelf-life analysis in ready-to-eat products (Bergis et al., 2021). However, in this study no significant changes in pH were observed for fresh-cut samples when compared to the beginning and the end of analysis, indicating other factors such as competition with other microorganisms and surface characteristics may have influenced the behavior of *L. monocytogenes*.

There are naturally occurring bacteria on the surface of fresh produce; the most commonly found microorganisms are aerobic bacteria, coliforms, and *Enterococcus faecium* (Johnston et al., 2006). The increase in MAB differs from the *L. monocytogenes* populations present in fresh-cut fruit samples when stored at 4°C, considering that this pathogen did not increase or decrease significantly in all fruit samples at this temperature. The population of these microorganisms may inhibit the growth of bacterial pathogens such as *L. monocytogenes* by outcompeting them for resources as stated by Maffei et al. (2016). Yeast and mold levels on pear and cantaloupe samples remained similar but increased significantly on papaya, pineapple and watermelon samples stored at 4°C. *L. monocytogenes* levels remained the same in these fruit samples stored at 4°C, possibly influenced by competition for nutrients with yeasts and molds. Gramisci et al. (2018) and Gensler et al. (2020) both demonstrated the antagonistic effect that the growth of yeast and mold can have on foodborne pathogens.

Temperature plays an important role on survival and attachment of microorganisms on produce matrices. Tian et al. (2012) observed no significant changes in *L. monocytogenes* population on romaine lettuce and iceberg lettuce at 4°C for 12 days. Chhetri et al. (2019) also observed a stable bacterial population on spinach surfaces up to 48 h under refrigerated temperature (4°C). However, the increase in the storage temperature to 15°C significantly increased the population after day 2, indicating the role of temperature on the growth of *L. monocytogenes* on produce matrices (Tian et al., 2012). Berrang et al. (1989) observed that Asparagus and broccoli supported the growth of *L. monocytogenes* at 15°C. Lokerse et al. (2016) determined an increase in the population from 1.20 to 1.50 log CFU/g at 7°C in kale samples. Martínez et al. (2015) reported similar results for broccoli and cauliflower with stable *L. monocytogenes* counts up to 8 log CFU/ml under the storage temperature of 10°C. For bacteria to grow, they must be able to first colonize the surfaces. *L. monocytogenes* is able to attach to produce surfaces; however, it fails to colonize on waxy outer surfaces (Fletcher et al., 2018). The variation on the growth and survival of the pathogen could also be attributed to other factors such as pH, available nutrients and antimicrobial substances which released due to cutting or manipulation (Kumar et al., 2015; Huang et al., 2019). The ability of *L. monocytogenes* in growing on the surfaces under these storage conditions may be in part due to increase in temperature and cut surfaces of the produce. The exposed internal surfaces might have enabled *L. monocytogenes* to internalize and colonize into produce (Fletcher et al., 2018). This finding indicated that the storage temperature abuse could increase the produce safety risk; however, the level of the risk could be dependent on the type of produce as well. Similarly, Kroft et al. (2022) found that *L. monocytogenes* population decreased on broccoli samples stored at 4°C by 1.00 log CFU/g. However, our results differed from them when it came to cauliflower considering they determined *L. monocytogenes* increased significantly in these samples at 10°C, and in bell pepper samples *L. monocytogenes* remained the same in 4, 10, 12, and 15°C storage temperature (Kroft et al., 2022).

The increase in MAB population in certain vegetables like kale may be connected to *L. monocytogenes* levels being reduced at this temperature, considering that the MAB successfully outcompeted *L. monocytogenes* on the surface of these samples. The increase in yeast and mold in broccoli samples were interesting since *L. monocytogenes* levels on similar samples were significantly decreased during the storage period. Yeast and mold populations on cauliflower stored at 4°C decreased significantly by 1.12 log CFU/g by the end of the study. Considering, *L. monocytogenes* levels also decreased significantly during the storage period indicate cauliflower does not provide a favorable environment for both *L. monocytogenes* and yeast and mold at 4°C. Broccoli, lettuce and bell peppers stored at 13°C had higher levels of yeast and mold however *L. monocytogenes* levels remained the same throughout the storage.

Surface characteristics of produce have the ability to influence the survival of microorganisms during storage. Both Gagliardi et al. (2003) and León-Félix et al. (2010) discussed that the contamination of melons and bell peppers with human pathogens during harvest and post-harvest may be influenced by their respective surfaces (Gagliardi et al., 2003; León-Félix et al., 2010). Stine et al. (2005) observed that cantaloupe surface better supported the survival for microorganisms including *E. coli*, and *Shigella* compared to lettuce or bell pepper surfaces. Our study also observed similar results with an increase in *L. monocytogenes* count during storage of cantaloupe rinds. However, there was a decreasing trend in the bacterial population on bell pepper rinds. The study of human pathogens’ growth on produce rinds is essential to assess the produce safety risks while processing fresh produce. During slicing of produce, contaminated pathogens on the rind may transfer into the edible portion of the fruits. Furthermore, cantaloupe is a good source of fructose, glucose, and sucrose, with soluble solids ranging from 8 to 14% (Lester and Dunlap, 1985). Leaching of these nutrients on the surface as a result of storage will favor the growth of microorganisms. In addition, crevices or cracks on rinds of cantaloupe can aid the formation of biofilms, considering bacteria can become firmly attached on these surfaces (Gautam et al., 2014). Our results emphasize this previous fact, considering how *L. monocytogenes* was able to survive on the external surface of cantaloupe. Conversely, at the end of the storage period of green bell pepper rinds, *L. monocytogenes* was reduced significantly due to lack of nutrients and of surfaces that facilitate attachment. This is in accordance with the study by Kroft et al. (2022), in which populations of *L. monocytogenes* on whole bell peppers decreased significantly during storage at 10 and 20°C (Kroft et al., 2022). It is important to highlight that the European Union Reference Laboratory for *Listeria monocytogenes* (EURL *Lm*) recommends an initial targeted contamination level of approximately 2 log CFU/g, which is near the initial contamination level in most fresh-cut or rind samples in this study. Results from challenge studies using similar contamination levels and temperature abuse conditions that reflects actual processing conditions would be valuable for risk assessment of *L. monocytogenes* in ready-to-eat fruits and vegetables (Bergis et al., 2021).

The ability of pathogens to transfer and internalize produce surfaces can be affected by the permeability of the surface. The evaluated contact angles of cantaloupe rinds using distilled water are similar to those obtained in our previous study in 2015. In this study the authors found the contact angle was 76.30° (Adhikari et al., 2015). Another study by Park and Kang (2017) evaluated the contact angle of the outer surface of bell peppers and obtained an average of 87.20°, both of these studies indicate that a distilled water contact angle >65° represents a hydrophobic surface which is in accordance to our findings with the contact angles of cantaloupe and bell pepper rinds (Park and Kang, 2017). The surface free energy determined for broccoli, bell peppers (fresh-cut and rinds) an cantaloupe rinds is similar to those obtained by Velásquez et al. (2011), in which they found the surface free energy of fruit epicarps to range between 37 and 44 mN/m. Hydrophobicity has been discussed in literature to have an influence in bacterial adhesion, considering that these microorganisms can be transported through liquids from one surface to another. However, Zhang et al. (2013) also highlighted the importance of evaluating hydrophobicity along with the surface free energy of the sample. While a surface may be hydrophilic, bacterial adhesion depends highly on Van der Waals interactions between the bacteria and the surface; therefore, low surface free energy makes it difficult for the bacteria to become firmly attached to the surface and form biofilms (Zhang et al., 2013). This is substantiated by past studies in which surface free energy values from 20 to 30 mN/m consistently exhibited less adhesion of bacteria such as *E. coli* O157:H7 and *Pseudomonas aeruginosa* (Callow and Fletcher, 1994; Becker, 1998; Zhao et al., 2004; Pereni et al., 2006).

Overall, our study indicated that the storage temperature of fruits and vegetables is a critical factor for the survival and growth of *L. monocytogenes,* and storage at refrigeration temperature is a key to reduce produce safety risks. The disparity in the bacterial growth and survival between the produce indicated that produce characteristics are other factors that must be considered while developing produce safety risk management strategies. The growth of *L. monocytogenes* on cantaloupe rinds at ambient temperature indicated that there is a need for a proper storage strategy for cantaloupe fruits. Additionally, the hydrophilic characteristics and high surface free energy of fresh-cut fruits indicate they are ideal environments for bacteria to contaminate. This study contributes to the present literature to understand better the growth of *L. monocytogenes* on produce surfaces. Further studies are needed to understand the interrelationship between comprehensive storage conditions and microbial survival on produce surfaces.

## Data availability statement

The original contributions presented in the study are included in the article/Supplementary material, further inquiries can be directed to the corresponding author.

## Author contributions

JM, EM, VS, and AA: conceptualization. JM and AA: investigation, resources, data curation, and writing – original draft preparation. JM, VS, EM, TG, JK, and AA: writing – review and editing. VS and AA: visualization, supervision, and project administration. AA: funding acquisition. All authors have read and agreed to the published version of the manuscript.

## Funding

This study was supported by the United States Department of Agriculture (USDA) National Institute of Food and Agriculture, Hatch project LAB94565 and USDA, LDAF Specialty Crop Block Grant Project 001891.

## Conflict of interest

The authors declare that the research was conducted in the absence of any commercial or financial relationships that could be construed as a potential conflict of interest.

## Publisher’s note

All claims expressed in this article are solely those of the authors and do not necessarily represent those of their affiliated organizations, or those of the publisher, the editors and the reviewers. Any product that may be evaluated in this article, or claim that may be made by its manufacturer, is not guaranteed or endorsed by the publisher.
