# Supplementary material for: Effect of storage temperature and produce type on the survival or growth of Listeria monocytogenes on peeled rinds and fresh-cut produce
Source: Front Microbiol. 2023 Jun 15;14:1151819. doi: 10.3389/fmicb.2023.1151819 (PMC10313384; doi:10.3389/fmicb.2023.1151819)
Supplement: Supplementary file 1 [file Table_1.DOCX]

Supplementary Material

# Supplementary Figures and Tables

## Supplementary Tables

SUPPLEMENTARY TABLE 1. pH measurement of fresh-cut fruit samples on the first and last day of storage at **(A)** 4˚C or **(B)** 13˚C storage temperature.

|  |  | Fruits* | | | | |
| --- | --- | --- | --- | --- | --- | --- |
| **Storage Temperature** | **Day** | Pear | Papaya | Pineapple | Watermelon | Cantaloupe |
| 4°C | 0 | 4.72±0.21 | 5.29±0.07 | 3.93±0.21 | 5.44±0.64 | 6.35±0.11 |
| 4°C | 6 | 4.66±0.38 | 5.32±0.10 | 3.88±0.35 | 5.47±0.60 | 6.26±0.16 |

|  |  | Fruits* | | | | |
| --- | --- | --- | --- | --- | --- | --- |
| **Storage Temperature** | **Day** | Pear | Papaya | Pineapple | Watermelon | Cantaloupe |
| 13°C | 0 | 4.47±0.19 | 5.33±0.08 | 3.44±0.11 | 5.18±0.19 | 6.28±0.12 |
| 13°C | 6 | 4.54±0.16 | 5.31±0.11 | 3.31±0.14 | 5.19±0.10 | 6.33±0.15 |

Analysis of each fruit at either 4˚C or 13˚C storage temperature was carried out in triplicate.

*No statistical differences (P>0.05) were found in any of the fruits when compared between Day 0 and 6, either 4˚C or 13˚C storage temperature.

SUPPLEMENTARY TABLE 2. pH measurement of fresh-cut vegetable samples on the first and last day of storage at **(A)** 4˚C or **(B)** 13˚C storage temperature.

|  |  | Vegetables* | | | | |
| --- | --- | --- | --- | --- | --- | --- |
| **Storage Temperature** | **Day** | Broccoli | Cauliflower | Lettuce | Kale | Green Bell Pepper |
| 4°C | 0 | 6.53±0.08 | 6.76±0.06 | 6.49±0.33 | 6.35±0.10 | 5.36±0.11 |
| 4°C | 6 | 6.39±0.12 | 6.59±0.12 | 6.45±0.20 | 6.47±0.10 | 5.43±0.18 |

|  |  | Vegetables* | | | | |
| --- | --- | --- | --- | --- | --- | --- |
| **Storage Temperature** | **Day** | Broccoli | Cauliflower | Lettuce | Kale | Green Bell Pepper |
| 13°C | 0 | 6.36±0.17 | 6.59±0.09 | 6.32±0.13 | 6.50±0.11 | 5.45±0.40 |
| 13°C | 6 | 6.33±0.10 | 6.56±0.21 | 6.43±0.11 | 6.48±0.13 | 4.93±0.23 |

Analysis of each vegetable at either 4˚C or 13˚C storage temperature was carried out in triplicate.

*No statistical differences (P>0.05) were found in any of the vegetables when compared between Day 0 and 6, either 4˚C or 13˚C storage temperature.
